# Fibrolytic efficiency of the large intestine microbiota may benefit running speed in French trotters: A pilot study

**DOI:** 10.14814/phy2.70110

**Published:** 2024-11-12

**Authors:** Maximilien Vasseur, Romuald Lepers, Nicolas Langevin, Samy Julliand, Pauline Grimm

**Affiliations:** ^1^ Institut National de la Santé et de la Recherche Médicale, Inserm UMR 1093, Cognition Action et Plasticité Sensorimotrice (CAPS), Faculty of Sport Sciences Université de Bourgogne Dijon France; ^2^ Lab to Field Dijon France; ^3^ Ecurie Hunter Valley Belfonds France

**Keywords:** fiber digestion, horse, metabolism, performance, short‐chain fatty acids

## Abstract

This pilot study sought to explore the contribution of the large intestine microbiota to energy metabolism and exercise performance through its ability to degrade fibers into short‐chain fatty acids (SCFAs). To investigate this, a correlational study was carried out on athlete horses under the same management conditions. Fecal microbiota diversity and composition, fibrolytic efficiency and SCFAs were analyzed. An incremental running test was carried out to estimate the maximal running speed (MRS) of the horses, and blood samples were taken to measure energy metabolism parameters. MRS was positively correlated with the efficiency of the fecal microbiota in degrading cellulose in vitro (*r* = 0.51; *p* = 0.02). The abundance of fibrolytic bacterial taxa was not associated with MRS, but functional inference analysis revealed a positive association between MRS and pathways potentially related to fibrolytic activity (*r* = 0.54; *p* = 0.07 and *r* = 0.56; *p* = 0.05 for butyrate metabolism and thiamine metabolism, respectively). In contrast, the metabolic pathway of starch degradation appeared negatively associated with MRS (*r* = −0.55; *p* = 0.06). The present findings suggest a potential contribution of the large intestine microbiota and dietary fibers digestion to exercise capacity in equine athletes.

## 1 INTRODUCTION

The large intestine microbiota plays a critical role in host physiology regarding energy harvest, homeostasis, and overall health (Sekirov et al., 2010). The various microorganisms are responsible for breaking down nutrients not previously digested by the host, particularly plant cell walls (cellulose and hemicelluloses), also known as fibers (den Besten et al., 2013). Short‐chain fatty acids (SCFAs) are the main products of fiber breakdown by bacteria, including acetate, propionate, and butyrate, present in an approximate molar ratio of 60:20:20 in the colon and stool of humans (den Besten et al., 2013). Acetate bypasses colonocytes and liver utilization, reaching arterial circulation and muscles where it can be used as an energetic substrate in the citrate cycle (Moffett et al., 2020; Van Hall et al., 2002). Propionate is a source for gluconeogenesis in the liver (Cummings et al., 1987), while butyrate serves as a fuel for colonocytes (Roediger, 1982). In humans, SCFAs appear to modulate blood glucose uptake independently of insulin (Han et al., 2014; Maruta et al., 2016) and muscle metabolism (Frampton et al., 2020). Recent findings suggest that large intestine microbiota activity could influence endurance capacities in mice model (Clauss et al., 2021). Germ‐free mice have reduced run time to exhaustion compared to specific pathogen‐free mice (Hsu et al., 2015; Kim et al., 2023), suggesting that the deprivation of large intestine microbiota is detrimental to muscle activity. Moreover, providing a diet low in dietary fermentable fibers reduces plasma and fecal SCFAs concentrations and impairs endurance capacities in healthy mice compared to mice fed a diet high in dietary fermentable fibers (Okamoto et al., 2019). Similarly, an antibiotic‐induced dysbiosis of the large intestine microbiota results in lower levels of cecal and plasma SCFAs and reduced endurance capacities of mice (Nay et al., 2019; Okamoto et al., 2019). Subcutaneous infusion of acetate after antibiotic treatment negates the impairment of endurance capacity (Okamoto et al., 2019). Several other studies also report that oral acetate supplementation reduces glycolysis and favors fatty acid utilization, resulting in limited glycogen depletion during exercise and enhanced glycogen replenishment after exercise (Fushimi & Sato, 2005; Pan et al., 2015). All these results highlight the importance of SCFAs derived from fiber digestion by the large intestine microbiota, as a source of energy for skeletal muscle and a regulatory contributor to metabolism. In humans, fecal analysis in athletes revealed higher concentrations of SCFAs compared to sedentary individuals, suggesting differences in large intestine microbiota activity based on fitness level (Barton et al., 2017).

The human athlete's large intestine microbiota has garnered significant attention in the last decade due to its impact on host physiology and potential role in sports performance (Deng et al., 2023). It is now evident that human athletes have a different large intestine microbiota from their sedentary counterparts in terms of composition, diversity, and function. For example, *Akkermansia muciniphila*, associated with enhanced intestinal barrier function and, reduced inflammation and metabolic disorders (Everard et al., 2013; Karlsson et al., 2012), appears more abundant in the athlete's microbiota (Bressa et al., 2017; Clarke et al., 2014). Fibrolytic genera such as *Faecalibacterium*, *Eubacterium,* and *Ruminococcus* are also more abundant in athletes (Fontana et al., 2023; Han et al., 2020; Xu et al., 2022). Higher microbial diversity has been observed in some studies comparing athletes to sedentary control (Clarke et al., 2014; Han et al., 2020), which could explain the greater range of metabolic functions carried out by athletes' large intestine microbiota (Fontana et al., 2023). Athlete's large intestine microbiota carries a greater abundance of metabolic functions associated with carbohydrates metabolism (pyruvate fermentation, glycolysis, pentose phosphate pathway, and citrate cycle), which provide energy substrates (Barton et al., 2017; Han et al., 2020; Liang et al., 2019). However, in the mentioned studies, athletes and sedentary controls have different types of diets, as high‐intensity exercise requires a specific diet to support energetic demands. Since diet is one of the most important factors modulating the microbiota, it is difficult to attribute the observed differences in microbiota solely to exercise levels (Hughes & Holscher, 2021). In addition, physical activity is also a known factor in modulating the large intestine microbiota (Aya et al., 2021). The control of these confounding factors is limited in human athletes, making it difficult to standardize the studied population. This limitation affects the ability to determine how the microbiota may impact performance. Moreover, the microbiota fibrolytic function of athletes has been little studied, despite evidence of the implication of SCFAs in modulating endurance capacities. Given the considerable inter‐individual variations in large intestine microbiota (Benson et al., 2010; Eckburg et al., 2005), it is likely that there are also variations in fibrolytic efficiency and SCFAs production among individuals, which may, consequently, influence endurance capacities.

In the present study, we used the equine model, which is appropriate for studying the relation between large intestine microbiota and exercise performance. First, the horse is a highly athletic mammal that can reach a maximal oxygen uptake of 220 mL/min/kg, more than twice that of a human athlete (Poole & Erickson, 2011). Second, the horse, as an herbivore, is an efficient large intestine fermenter of fibers (Wunderlich et al., 2023), and when fed a high‐fiber diet, SCFAs can cover 60 to 80% of the daily energy requirements (Vermorel & Martin‐Rosset, 1997) compared to 5 to 15% in humans (Bergman, 1990; McNeil, 1984). Finally, the horse is a model where standardized management can be applied within the same stable, limiting the impact of confounding factors such as diet and exercise. Several studies have already focused on the relation between large intestine microbiota and exercise performance in equine athletes. Consistent with findings in humans, elite racehorses have greater microbial diversity and abundance of fibrolytic taxa such as Lachnospiraceae, *Prevotella*, and *Ruminococcus*, compared to non‐elite horses of the same breed (Park et al., 2024; Li et al., 2023).

This pilot study investigated the correlation between large intestine microbiota, energy metabolism, and running performance. A global microbiota composition, function, and activity analysis would enhance our comprehension of fibrolytic efficiency and its significance in exercise capacities here measured by running speed during an incremental running test. The study was conducted on a homogeneous population of equine athletes from a single training center with a standardized diet and training management. It was hypothesized that a more efficient microbiota at breaking down dietary fibers would provide beneficial energy metabolites to the horse, which could contribute to greater running speed.

## 2 MATERIALS AND METHODS

### 2.1 Animals, management, and design

This study was carried out at the Hunter Valley stable in France, which breeds and trains top‐level French Trotters. We recruited 26 horses (mean ± SD: 2.0 ± 0.1 years old, 426 ± 30 kg, 4.2 ± 0.8 score on Henneke body condition score grid (Henneke et al., 1983)) from their generation of 2‐year‐old horses to conduct a correlational study. This number of horses appeared to be sufficient to emphasize correlations with a threshold of significance of 10% (22 individuals required for *r* = 0.5 and power = 0.8). This study was conducted under the agreement of the Plateforme NSP Animal Welfare Body (EU0437, #PNSP2201). This protocol was approved by the trainer, and written consent from horse owners was obtained before the sample collection. All procedures were done with respect to animal welfare by an equine veterinary practitioner from the training center.

The studied cohort comprised 16 males and 10 females. The horses began training at 18 months old to qualify for racing in France at 2 years of age. Measurements were taken 6 months after training began and before their qualifying race. Horses included in the study were healthy and considered fit to follow the current training program by the trainer. All horses were up to date with their vaccination program and were last dewormed at least 1 month prior to the study as it can alter large intestine microbiota (Walshe et al., 2019).

Horse management was followed during the month before the measurements. Three days a week, horses were trained in the morning. Typical training sessions consisted of four running intervals at an average speed of 10.5 ± 0.5 m.s^−1^ separated by a recovery time of 2 to 4 min walking or at slow trot. The workload was considered as very heavy for horses as the mean heart rate over the entire exercise bout was higher than 150 bpm (National Research Council, 1989). Horses were housed in paddocks of two (males) or fours (females), and during training days, they were stabled in individual boxes on straw bedding. Horses had free access to grass hay, water, and mineral salt (Salt Lick Advantage, Equisalt, Sweden) in boxes and paddocks. For each horse individually, a pelleted concentrate (Groov Protein, Krafft, Sweden) was fed in the morning and in the evening (2.2 kg per meal) and a muesli concentrate (High Energy Muesli, Krafft, Sweden) was fed at midday (2.0 kg per meal). No feed refusals were observed. Based on the frequency of hay bale distribution in the paddocks and the quantity consumed in the boxes, the individual daily hay consumption was estimated to be 7.0 kg. Energy and protein requirements were met according to nutrient recommendations, considering a very heavy weekly workload (National Research Council, 1989). Daily nutrient intakes are presented in Table 1 based on chemical analysis (Equine complete analysis, DairyOne, Ithaca, NY, USA).

**TABLE 1:** Feed composition and daily ration (as fed).

	Hay	Pelleted feed	Muesli feed
Dry matter %	92.0	89.3	87.9
Digestible energy, Mcal.kg^−1^	2.0	2.6	3.1
Crude protein, g.kg^−1^	79.7	137.0	137.0
Neutral Detergent Fiber, g.kg^−1^	530.1	286.0	169.0
Acid Detergent Fiber, g.kg^−1^	359.6	161.0	122.0
Starch, g.kg^−1^	25.7	189.0	260.0
Simple sugars, g.kg^−1^	62.0	61.0	61.0
Fat, g.kg^−1^	19.7	47.2	85.4
Daily intake, kg.day^−1^	7.0^a^	4.4	2.2

^a^ Estimated quantity.

Feces were collected on each horse to measure fecal microbiota composition, diversity, and functions. Anaerobic culture techniques were also used to determine fibrolytic efficiency and count cellulolytic bacteria. For logistical reasons, horses performed an incremental exercise test to assess their energy metabolism and physical performance 2 weeks after fecal microbiota analyses. As diet and training were the same over at least 1 month before fecal sample collection and the 2‐week interval before the exercise test, we assumed that large intestine microbiota composition and exercise capacity would remain stable.

### 2.2 Fecal sample collection and analysis

Feces were collected by rectal grab after the morning meal and before training. Fecal samples were collected in sterile microtubes and stored at −20°C during the trial period, followed by storage at −80°C in our laboratory for further bacterial 16S rRNA gene sequencing analysis. Two covered flasks were filled to the maximum capacity with feces to avoid the presence of oxygen, and feces were immediately used for the in vitro fermentation test and cellulolytic bacteria inoculation. Ten grams of feces were dried at 70°C for 48 h to determine dry matter content. The rest of the feces was filtered through a 100 μm filter. The pH of the liquid phase was determined (Eutech pH 700, Thermo Scientific, Waltham, USA) and aliquots were collected in microtubes (0.1 mL mixture of 4.25% H_3_PO_4_ + 1% HgCl_2_ was added as a preservative) and kept at −20°C for SCFAs analysis.

#### 2.2.1 Bacterial 16S rRNA gene sequencing analysis

Total DNA was extracted from 0.25 g of feces, as previously described by Yu and Morrison (Yu & Morrison, 2004), using the QIAamp Fast DNA Stool Mini Kit (cat. n° 51,604, Qiagen, Hilden, Germany). The quantity and purity of the DNA obtained were assessed by spectrophotometry (NanoPhotometer® C40, Implen, München, Germany). A first polymerase chain reaction (PCR) was performed to amplify the V3‐V4 hypervariable region of the 16S rRNA gene as described previously (Grimm et al., 2020), except that the Taq polymerase (2 U/μl IPROOF HF DNA Polymerase, cat n° 1,725,302, Bio‐Rad, California, USA) and the conditions of the PCR (1 cycle at 98°C during 1 min, followed by 30 × [98°C during 1 min, 65°C during 1 min and 72°C during 1 min]) were modified. The sequencing analysis of the 16S rRNA amplicons was performed externally (Genotoul Bioinformatics Platform, Toulouse, France). Briefly, a second PCR was carried out after amplicons purification and the PCR products obtained were sequenced using an Illumina MiSeq run of 250‐paired ends, according to the manufacturer's instructions (Illumina Inc., San Diego, CA, United States).

FROGS (Find Rapidly OTU with Galaxy Solution) metabarcoding pipeline on the Galaxy server was used to perform bioinformatics analysis (Escudié et al., 2018). The reads were merged and cleaned for sequences without primers or out of range (<400 or >580 base pair). SWARM technique was used to regroup sequences into clusters (distance = 1) and chimeric sequences were removed. Clusters being present in less than two samples and with an abundance <0.005% were eliminated. The remaining clusters were sorted into amplicon single variants (ASVs) and aligned to the silva138.1 16S data base using the BLAST method. Richness (number of ASVs and Chao1) and diversity (Shannon and inverse Simpson) indices were calculated from the abundance table. The relative abundance of each ASV, bacterial phylum, family and genera was calculated in relation to the total number of sequences per sample.

To study the potential metabolic functions of the identified ASVs, the PICRUSt2 tool (Douglas et al., 2020) was used with the KEGG (Kyoto Encyclopedia of Genes and Genomes) database. Each ASV was placed on a phylogenetic tree, and marker copy numbers were determined in each ASV. Affiliations with a NSTI (Nearest Sequenced Taxon Index) >0.5 and an identity and coverage percentage < 90% were eliminated. KEGG database was then used to predict each sample's function and pathway abundances.

#### 2.2.2 Cellulolytic bacteria counting

Fresh fecal samples were used to prepare a ten‐fold dilution series from 10^−5^ to 10^−8^ under O_2_‐free CO_2_ in an anaerobic mineral solution (Bryant & Burkey, 1953). Serial dilutions were inoculated in roll tubes containing a complex liquid medium (Julliand et al., 1999) with a strip of filter paper (Whatman n°1) as the sole cellulose substrate, and cellulolytic bacteria were cultured for 14 days at 38°C (Halliwell & Bryant, 1963). The most probable number (MPN) of cellulolytic bacteria per gram of feces was determined using the method of Mac Grady (Clarke & Owens, 1983) and this concentration was converted into base 10 logarithms.

#### 2.2.3 Determination of fecal fermentation end‐products

Fecal acetate, propionate, butyrate, iso‐butyrate, valerate and iso‐valerate concentrations were analyzed by gas–liquid chromatography (Clarus500, PerkinElmer, Courtaboeuf, France) as described by Jouany et al. (Jouany, 2024). Each SCFA concentration was expressed in mmol/l of filtrate and as a proportion of total SCFAs concentration. Additionally, D‐ and L‐lactic acid concentrations were analyzed by spectrophotometry at 340 nm (AMR‐100, Allsheng, Hangzhou, China) using an enzymatic colorimetric kit (D−/L‐lactic acid (rapid) Assay Kit, K‐DLATE, Megazyme Wicklow, Ireland) as described previously by Grimm et al. (Grimm et al., 2017). Total lactate concentration was also determined.

#### 2.2.4 In vitro fermentation test

Fibrolytic efficiency was measured individually through an in vitro fermentation test under anaerobic conditions, as described by Theodorou et al. (Theodorou et al., 1994). For each horse, 15 g of feces were added to a bottle containing 150 mL of culture medium (Lowe et al., 1987), and shaken for 3 min to separate microorganisms from solid particles. The liquid phase was filtered in a 100 μm filter. Twenty milliliter of each fecal inoculum were added into 5 bottles of 200 mL containing 80 mL of culture medium. Two bottles, identified as inoculum blank, contained only culture medium and the fecal inoculum, and three bottles also contained 2 g of dried hay as substrate. Hay was the same as that given to the horses in their diet; it was previously washed and grounded (<1 mm) to mimic mastication and foregut digestion. The bottles were incubated anaerobically at 38°C in a water shaker bath for 48 h. Gas production (GP) was manually measured in each bottle after 6, 12, 24, 30, 36 and 48 h of fermentation using a pressure transducer (HD2114, Delta OHM, Italy) and then released to zero after each measure. After 48 h of fermentation, bottles were placed on ice to arrest fermentation, and the content was filtered in a 38 μm filter. pH was measured in the filtrate and substrate residues were dried at 70°C for 48 h and analyzed (Equine complete analysis, DairyOne, Ithaca, NY, USA) to determine DM (DM_d_), cellulose (cellulose_d_), and hemicelluloses (hemicelluloses_d_) disappearances.

### 2.3 Maximal running test and blood metabolites determination

#### 2.3.1 Maximal running test protocol

Exercise capacity of each horse was measured through a maximal running field test performed in the morning. The horses were fed 1.5 h before the commencement of the test and were then harnessed with their usual equipment, including a sulky (wheeled cart where is seating the driver). The test was carried out on a 1000 m circular track located in the training center with two horses running on opposite sides to avoid influencing each other. It was adapted to the age of the horses (Allen et al., 2016) and consisted of a first phase of warm‐up (15 min at 5.5 m.s^−1^), then a stepwise increase in velocity (9.7 m.s^−1^, 11.1 m.s^−1^ and maximal velocity). Each step lasted 3 min, separated by one‐min recovery walking. During the last step, the drivers were instructed to reach the horses' maximum speed and to maintain it for as long as possible. The maximal running speed (MRS) was defined as the maximal speed maintained for at least 1 min during the last step. The test was stopped when the horse was unable to maintain the speed. After the final step, the horse returned to the stables, where they had 15 min of recovery at 0.8 m.s^−1^ in an automatic walker. Heart rate and speed were measured during the test using a heart rate monitor (Equimètre, Arioneo, France) equipped with a GPS which provides readings every second.

#### 2.3.2 Blood sample collection and analysis

Blood samples were collected at rest immediately before the running test (T_0_), 5 min after the end of the test at the stables (T_5_) and after the 15‐min recovery in the walker (T_20_). Blood was collected in a dry tube at the three‐time points to measure, using portable analyzers, glycemia (Accu Check Performa, EAN: 4015630063628, Roche Diabetes Care GmbH, Germany) and lactatemia (LactatePro2, EAN: 4987486483038, Arkray, Japan). Blood was also collected in lithium‐heparinized tubes and then centrifugated at 1500 g for 10 min to collect plasma. Plasma samples were stored at −20°C for later analysis of plasma acetate and non‐esterified fatty acids (NEFA). As previously described, plasma acetate concentration was measured using an enzymatic kit (Acetic Acid Assay Kit, K‐ACET, Megazyme, Wicklow, Ireland) (Laroche et al., 2024). NEFA was analyzed externally (Iodolab, Grézieu‐la‐Varenne, France) using endpoint enzyme assay (NEFA FS, cat n° 15,781, Diasys, Germany).

### 2.4 Statistical analysis

#### 2.4.1 Fitting models for in vitro gas production

After correction for GP for each bottle by subtracting the GP measured in the inoculum blank bottles at each time point, individual GP kinetic was adjusted using a proc. NLIN on SAS software (SAS® Studio, SAS Institute Inc., Cary, NC, USA) to the Mitscherlich equation:

$$\mathrm{GP}=A\times \left(1-e^{-n\times \text{Time}}\right)$$

In this equation, A is the asymptotic gas production (mbar) and n (h^−1^) is the fractional rate of fermentation. This equation was chosen as it represents typical gas production profile (Powell et al., 2020). Time to produce half of A (T_half_A) was calculated from this equation. The area under the curve (AUC) was also determined from the individual gas production curves.

#### 2.4.2 Correlation analysis

Statistics were performed on Rstudio software (R: The R Project for Statistical Computing, 2024). Normal distribution of the variables was verified using the Shapiro–Wilk test. Pearson correlation test or Spearman rank test, when normality was not verified, was performed to assess the relation between MRS and the different variables measured. Variables were clustered in groups (fecal parameters, bacterial taxonomic ranks, predicted metabolic pathways, in vitro parameters, and blood metabolites) to apply a false discovery rate (FDR) correction on *p*‐values obtained using the Benjamini‐Hochberg method. Correlation analysis of fecal and in vitro parameters with blood parameters was also performed. As this study was exploratory, and conducted on a small number of individuals, a significance threshold of *p* < 0.1 after FDR correction was considered.

## 3 RESULTS

Of the 26 horses initially recruited, 5 were excluded because of injury or antibiotic treatment during the month preceding the measurements. All measurements have been performed on the remaining 21 horses and physiological and exercise measurements are presented in Table 2. No correlation was found between MRS and age (*r* = −0.08; *p* = 0.73), body mass (*r* = −0.10; *p* = 0.66) and BCS (*r* = −0.19; *p* = 0.44). There was no difference between both sexes for all variables measured (*p* > 0.3).

**TABLE 2:** Individual characteristics and exercise measurements of the 21 horses considered.

	Variables	Mean ± SD	Range (min–max)
Individual characteristics	Age (years)	2.0 ± 0.1	1.8–2.3
	Body mass (kg)	424.2 ± 28.2	343–463
	BCS (/9)	4.2 ± 0.6	3.2–5.3
Exercise measurements	MRS (m.s^−1^)	11.8 ± 0.3	11.2–12.6
	HR_max_ (bpm)	244 ± 5	230–252

Abbreviations: BCS, body condition score; HR_max_, maximal heart rate during exercise; MRS, maximal running speed.

### 3.1 Correlation between fecal bacterial diversity, composition and exercise performance

From the 21 fecal samples, 548,888 sequences of 16S rDNA V3‐V4 regions were obtained. After quality filtering during the bioinformatic process, the number of sequences was reduced to 207,100 sequences (9862 ± 1889 sequences per sample). From these sequences, 2315 ASVs were identified and were affiliated to 11 phyla, 15 classes, 28 orders, 49 families, and 90 genera. Firmicutes (62.0 ± 6.0%), Bacteroidota (33.4 ± 6.2%), Spirochaetota (1.7 ± 1.0%) and Actinobacteria (1.2 ± 0.7%) were the dominant phyla identified. Mean relative abundances of unidentified ASVs for each taxonomic level were: 0.04 ± 0.04% at the order level, 2.30 ± 1.24% at the family level, 30.52 ± 6.65% at the genus level and 94.34 ± 1.09% at the species level. From these, taxonomic affiliations at the phylum, family, and genera levels with relative abundance greater than 0.1% were correlated with MRS. No significant correlation was found between MRS and the relative abundance of identified families or genera (*p* > 0.18; Supplementary Table S1).

MRS was negatively correlated to fecal bacterial richness indexes but was not significantly correlated to diversity indexes (Table 3).

**TABLE 3:** Correlations between fecal bacterial richness and diversity indices and MRS (*n* = 21).

Diversity index	*r*	*p*‐value	Mean ± SD
Observed ASVs number	−0.39	0.10	1294 ± 161
Chao1 richness	−0.41	0.09	1630 ± 167
Shannon index	−0.27	0.25	6.0 ± 1.0
Inverse Simpson index	−0.26	0.25	167 ± 70

### 3.2 Correlation between metabolic pathways and exercise performance

**FIGURE 1:**
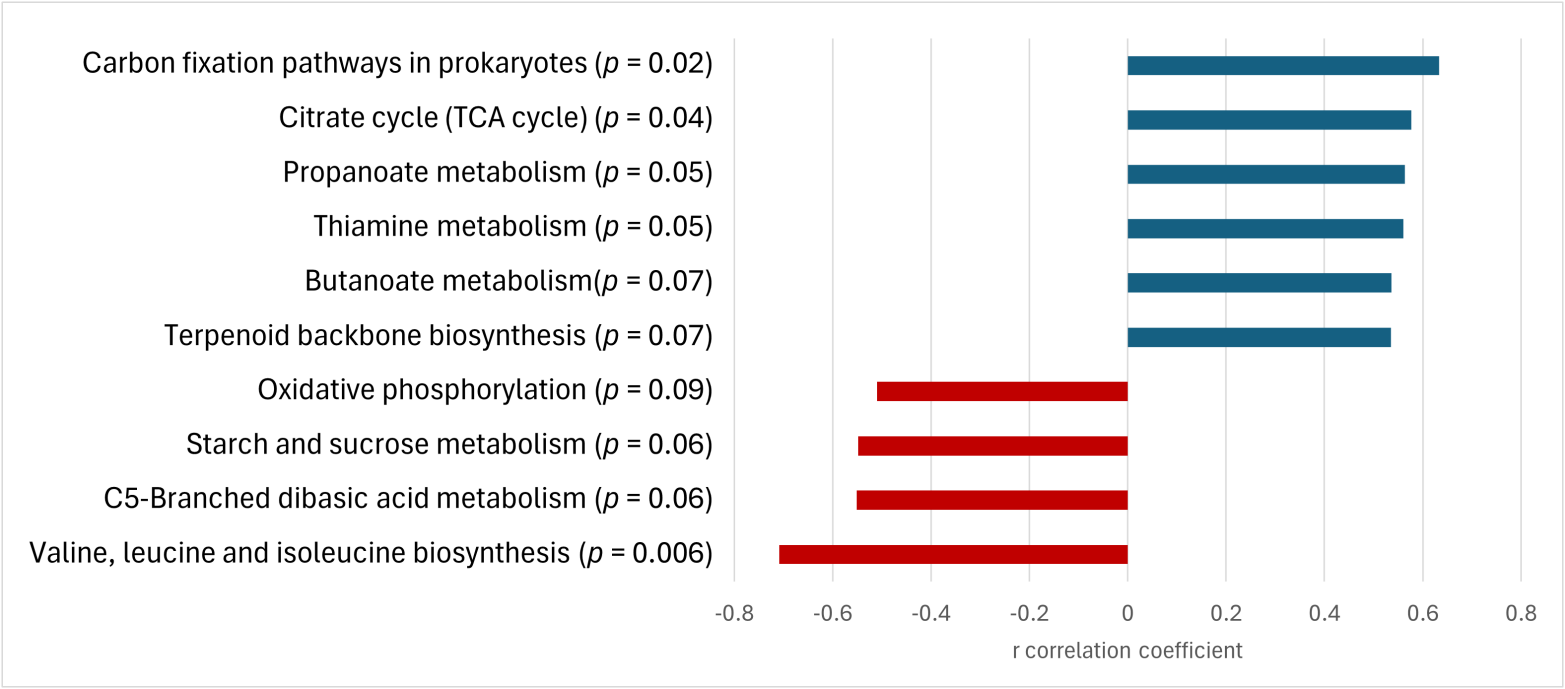
Significant correlations coefficient between MRS and predicted metabolic pathways from the KEGG database (*n* = 21; *p* < 0.1).

Identification of metabolic pathways based on 16S rRNA gene sequences allows to predict potential functions carried out by individual fecal microbiota. Of the 122 metabolic pathways identified, 89 had a relative abundance greater than 0.1% (Supplementary Table S2) and 11 of these were correlated to MRS (*p* < 0.10; Figure 1). MRS was positively correlated to pathways of butanoate metabolism, propanoate metabolism, citrate cycle, carbon fixation, thiamine metabolism and terpenoid metabolism. On the contrary, reduced running performance during exercise was associated with a greater abundance of pathways of C5‐branched dibasic acid metabolism, BCAA biosynthesis, starch and sucrose metabolism, and oxidative phosphorylation.

### 3.3 Correlation between fecal, fibrolytic efficiency, and blood metabolites measurements and exercise performance

**FIGURE 2:**
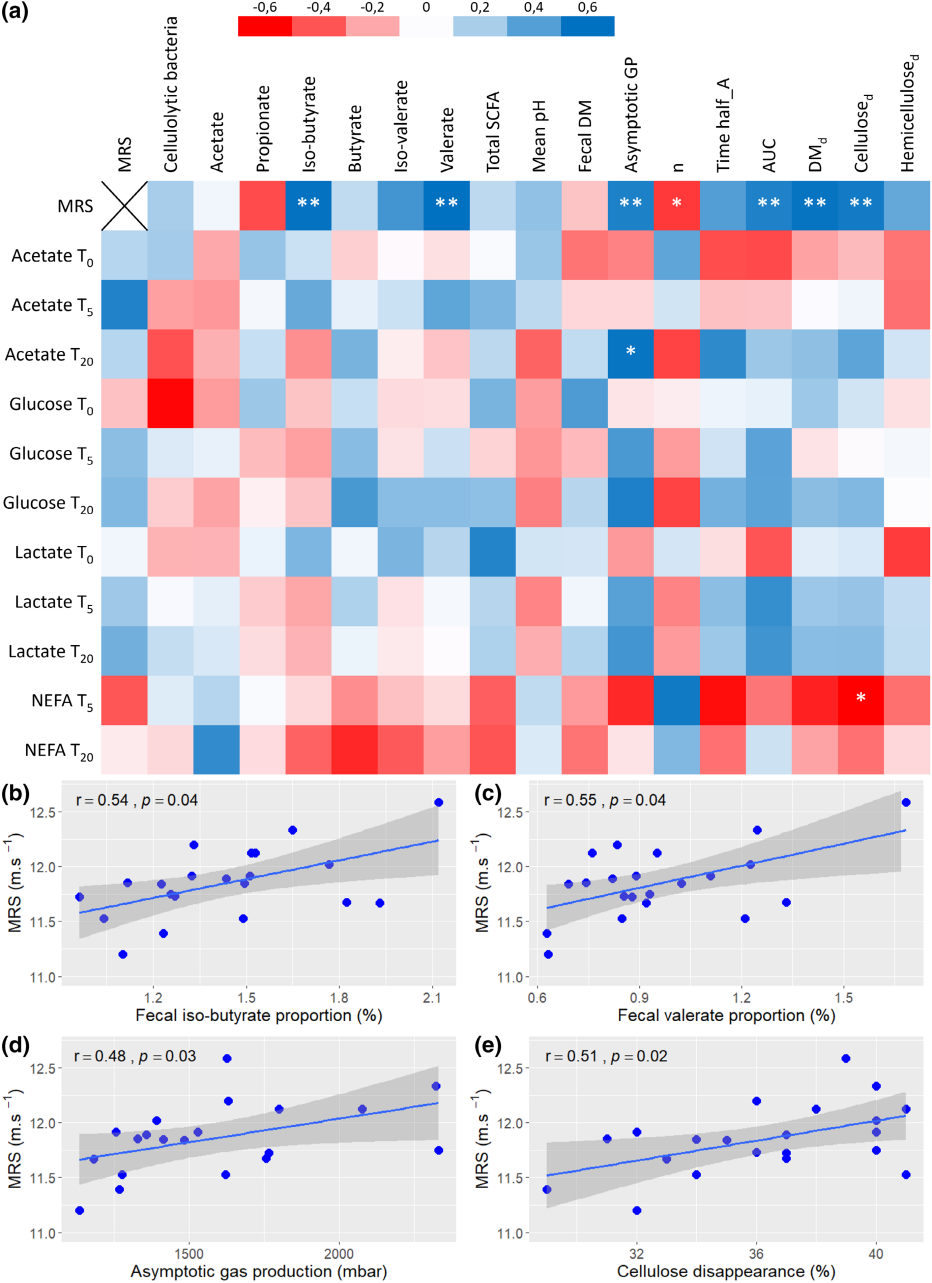
Correlations between physiological characteristics and MRS (*n* = 21). (a) Correlation matrix of fecal, in vitro, blood and performance variables. (b) Scatterplot of correlation between MRS and fecal iso‐butyrate proportion. (c) Scatterplot of correlation between MRS and fecal valerate proportion. (d) Scatterplot of correlation between MRS and asymptotic gas production. (e) Scatterplot of correlation between MRS and in vitro cellulose disappearance. **p < 0.05; *0.1 < *p* < 0.05; blanks: *p* > 0.1; DM, dry matter; GP, gas production; MRS, maximal running speed; SCFA, short‐chain fatty acids; NEFA, non esterified fatty acids.

Mean values of the different parameters measured are presented in Table 4. Correlation analysis (Figure 2a) revealed a positive relation between MRS and fecal proportion of iso‐butyrate (*r* = 0.54; *p* = 0.04; Figure 2b) and valerate (*r* = 0.55; *p* = 0.04; Figure 2c). No correlation was observed between the other SCFAs proportions and MRS. Greater MRS was associated with higher asymptotic gas production during the in vitro fermentation test (*r* = 0.48; *p* = 0.03; Figure 2d) and with lower fractional fermentation rate (*r* = −0.42; *p* = 0.07). Substrate dry matter and cellulose disappearances were positively correlated with MRS (*r* = 0.53; *p* = 0.02 and *r* = 0.51; *p* = 0.02 respectively; Figure 2e). Asymptotic gas production was positively correlated with plasma acetate concentration after exercise recovery (*r* = 0.53; *p* = 0.08), while cellulose disappearance was inversely correlated with plasma NEFA concentration after exercise (*r* = −0.55; *p* = 0.07).

**TABLE 4:** Mean values of parameters measured on the 21 horses.

	Variables		Mean ± SD
Fecal measurements	Cellulolytics (log_10_ MPN.g^−1^)		6.8 ± 0.7
	Acetate (%)		74.1 ± 2.2
	Propionate (%)		15.4 ± 2.1
	Butyrate (%)		6.1 ± 1.1
	Valerate (%)		1.0 ± 0.3
	Iso‐butyrate (%)		1.4 ± 0.3
	Iso‐valerate (%)		1.9 ± 0.6
	Total SCFAs (mmol.L^−1^)		125.0 ± 27.3
	D‐Lactate (mmol.L^−1^)		0.5 ± 0.2
	L‐Lactate (mmol.L^−1^)		0.4 ± 0.1
	Total Lactate (mmol.L^−1^)		1.0 ± 0.2
	pH		6.2 ± 0.6
	Dry Matter (%)		25.3 ± 3.3
In vitro measurements	A (mbar)		1577 ± 349
	n (.h^−1^)		0.024 ± 0.006
	T_half_A (h)		30.1 ± 7.4
	AUC		11,576 ± 1133
	DM_d_ (%)		19.0 ± 2.8
	Cellulose_d_ (%)		36.2 ± 3.4
	Hemicelluloses_d_ (%)		33.6 ± 4.2
Blood metabolites measurements	Acetate (mmol.L^−1^)	T_0_	0.96 ± 0.34
		T_5_	1.86 ± 0.47
		T_20_	1.40 ± 0.32
	Glucose (mg.dL^−1^)	T_0_	118.0 ± 18.6
		T_5_	187.1 ± 28.6
		T_20_	155.0 ± 28.5
	Lactate (mmol.L^−1^)	T_0_	1.4 ± 0.2
		T_5_	16.4 ± 6.1
		T_20_	9.4 ± 5.0
	NEFA (mmol.L^−1^)	T_0_	0.10 ± 0.00
		T_5_	0.32 ± 0.12
		T_20_	0.33 ± 0.13

Abbreviations: A, Asymptotic gas production; AUC, area under the curve; Cellulose_d_, cellulose disappearance; DM_d_, dry matter disappearance; Hemicelluloses_d_, hemicelluloses disappearance; n, fractional rate of fermentation; NEFA, non‐esterified fatty acid; T_half_A, time to produce half of A.

## 4 DISCUSSION

This study explored the correlations between large intestine microbiota, energy metabolism and exercise capacity in a population of equine athletes. Fiber degradation by the fibrolytic microbiota could provide substantial quantities of SCFAs to the horse to be used as an extra source of energy during exercise. To assess the fibrolytic efficiency of each horse's large intestine microbiota, we used a global approach combining the sequencing technic and functional inference, the enumeration of cellulolytic bacteria by culture, dosing of fermentation end‐products and an in vitro test of hay degradation. To the best of our knowledge, this is one of the first studies to have employed a cohort of individual athletes in a standardized manner with respect to diet and training, thus focusing on interindividual variations of the large intestine microbiota.

### 4.1 The fibrolytic activity of the large intestine microbiota appears to be positively associated with exercise capacity in horses

Our results suggest that while the composition of the large intestine microbiota does not appear to play a major role in exercise capacity, its fibrolytic efficiency seems to benefit exercise capacity. In this sense, we found that the gas production and the rate of cellulose disappearance measured during the in vitro test were positively associated with the MRS of horses measured during the running test, suggesting a better digestion of cellulose in the more athletic horses. It would be assumed that these horses possess a more abundant microbial population with cellulolytic activity. This was not confirmed by our result, which did not reveal a correlation between MRS and cellulolytic bacteria counting or taxonomic analysis. For example, *Ruminococcus* and *Fibrobacter*, which have been identified as important fibrolytic taxa in horses (Biddle, Stewart, et al., 2013) and humans (Wedekind et al., 1988), were not related to MRS in our experiment. This suggests that MRS is not associated with microbial composition, but rather with enhanced cellulolytic activity. This hypothesis can be supported by the results of the functional inference analysis, as some metabolic pathways potentially related to fibrolytic activity (butyrate metabolism and thiamine metabolism which is a cofactor of pyruvate dehydrogenase leading to acetyl‐CoA production, which is converted to acetate) (Froidurot & Julliand, 2022; Zhang et al., 2021) were positively correlated with MRS. The greater abundance of fibrolytic pathways was observed in endurance horses with high cardiovascular fitness (Mach et al., 2022) and racehorses (Park et al., 2024; Li et al., 2023) compared to horses with a low to moderate level of performance, suggesting improved acetate and butyrate synthesis in horses with high exercise capacities. A positive correlation between fecal butyrate concentration and VO_2max_ was also observed in a cohort of healthy human individuals (Estaki et al., 2016). However, this relation between MRS and fecal butyrate or fecal acetate was not shown in our study. In the context of studying athletes, feces is the only accessible matrix to assess the composition and activity of the large intestine microbiota, which adds a degree of complexity to the interpretation of results as feces also provide insight into the unabsorbed metabolites (Grimm et al., 2017).

Studies in horses have suggested that greater fibrolytic activity, induced by a high‐fiber diet, would increase the level of circulating acetate at rest (Jansson & Lindberg, 2012; Jensen et al., 2016). Greater circulating acetate has been observed to increase acetate contribution to muscle oxidation at rest (Pethick et al., 1993), which can limit the utilization of other energy sources such as glycogen (Karlsson et al., 2002) and delay lactate threshold (Jansson & Lindberg, 2012). Its importance as a substrate for energy metabolism in mammals has been confirmed by Okamoto et al. (Okamoto et al., 2019), who demonstrated that the impaired endurance capacities of mice treated with antibiotics were negated by subcutaneous acetate infusion. While we found no correlation between blood acetate concentration at rest and MRS, we observed that greater circulating acetate after recovery in horses was correlated with higher fibrolytic efficiency, suggesting greater availability when acetate is produced in high amounts in the large intestine. We hypothesize that this mechanism could have occurred during the recovery between each increment of the running test, allowing greater blood acetate replenishment in horses with high fibrolytic efficiency. This could have conferred additional substrate, limiting the depletion of glycogen for later utilization at high intensity (Martin et al., 2023). Blood kinetics would be necessary to validate this hypothesis.

### 4.2 The diversity and composition of the microbiota can vary due to athlete management

In contrast to our findings, some studies have suggested that the composition and activity of athletes' microbiota may differ from those of sedentary individuals. Higher fecal SCFA concentrations (Barton et al., 2017) and, greater abundance of beneficial bacteria such as *Akkermansia muciniphila* (Bressa et al., 2017; Clarke et al., 2014) and fibrolytic taxa such as *Faecalibacterium, Eubacterium* and *Ruminococcus* (Fontana et al., 2023; Han et al., 2020; Xu et al., 2022) have been observed in athletes compared to sedentary individuals. These observations could be attributed primarily to the increased physical activity and the dietary requirements of athletes, which necessitate a higher intake of energy and protein (Hughes & Holscher, 2021). In a standardized population of healthy adult individuals, Estaki et al. (Estaki et al., 2016) did not observed specific taxa correlated with VO_2max_, confirming our similar observation on equine athletes. While our results demonstrated an inverse relation between species richness and MRS, it was reported that species richness was higher in racehorses than in sedentary individuals (Park et al., 2024) and positively correlated with VO_2max_ in healthy humans (Estaki et al., 2016). It was established that depending on the frequency and intensity of exercise, the response in human large intestine microbiota composition and diversity may vary (Bonomini‐Gnutzmann et al., 2022). Physical activity has a hormetic effect on intestinal health (Mailing et al., 2019) and repeated vigorous exercise could be detrimental to large intestine homeostasis due to ischemia (Moses, 2005). This highlights the importance of standardized exercise intensity to decipher the impact of microbiota on individual exercise capacity. Furthermore, this study was conducted on 2‐year‐old French trotters which had not yet reached adult morphology and had only undergone 6 months of training, meaning they were not at peak performance levels. In contrast, the cited studies are based on adult individuals and athletes at their full performance potential. It would be interesting to assess how these relationships evolve with age.

### 4.3 Individual variability in ability to digest starch can influence fibrolytic efficiency and protein degradation

Physical activity and dietary composition, which appear as potential confounding factors when comparing athletes to sedentary individuals were standardized in our population. All horses were fed the same amounts of concentrate, but the amount of the different nutrients reaching the large intestine could vary between individuals, potentially altering the microbial fermentation profiles in the large intestine. The large intestine is the main digestive compartment for fiber degradation by the microbiota in horses, but the functional diversity of the large intestine microbiota allows it to degrade other previously undigested substrates reaching the large intestine, such as starch and protein. Horses have a limited capacity for enzymatic digestion of starch in the small intestine. Thus, feeding performance horses starch over 1 g.kg^−1^ BW.meal^−1^ can result in substantial quantity of starch reaching the large intestine where it is rapidly fermented (Julliand et al., 2006). In our study, the horses' diet provided a mean (± SD) starch content of 3.3 ± 0.2 g.kg^−1^ BW.day^−1^, with 1.0 ± 0.1 g.kg^−1^BW.meal^−1^ in the morning and evening meals and 1.4 ± 0.1 g.kg^−1^BW.meal^−1^ at midday. This amount can result in a proportion of starch entering the large intestine. We observed a negative correlation between starch and sucrose metabolic pathways, in vitro fractional rate of fermentation and MRS. A higher fractional fermentation rate would correspond to faster fermentation, particularly of starch and sugars, which are rapidly degraded. Horses with low MRS might, therefore, have higher microbial amylolytic activity, potentially due to lower antecaecal starch digestion. However, the abundances of bacterial genera identified as starch utilizer in horses, such as Streptococcus (Al Jassim et al., 2005), were not associated to lower MRS. Starch fermentation in the large intestine leads to a rapid drop in pH due to lactate production, which is detrimental for fibrolytic bacteria and their fiber‐degrading activity (Wunderlich et al., 2023). Lactate can be converted to SCFAs (mainly propionate) by lactate‐utilizing bacteria (Biddle, Black, & Blanchard, 2013), limiting intestinal acidosis due to lactate accumulation (Wang et al., 2020). Propionate can then be converted into valerate, which can benefit intestinal health and tissue integrity (Li et al., 2020). In this sense, we found that the pathway of propionate metabolism and the fecal proportion of valerate were positively correlated with MRS, suggesting a greater capacity to convert lactate to propionate in the best horses. This suggests that horses with a microbiota capable of degrading lactate to propionate and then to valerate could benefit from a limited reduction in pH, which could favor fiber degradation, and thus exercise capacity. Efficient fiber degradation can induce lower substrate availability in the distal part of the large intestine where pH is close to neutrality, favoring protein degradation (Cummings & Macfarlane, 1991). Branched chain fatty acids (BCFAs), iso‐butyrate and iso‐valerate, originate from branched chain amino acids (BCAAs) degradation and are markers of proteolytic activity (Smith & Macfarlane, 1998). We observed a positive correlation between fecal iso‐butyrate proportion and MRS, suggesting greater proteolytic activity in horses with the highest exercise capacities. These BCFAs could benefit fibrolytic microorganisms activity and fiber degradation, thus leading to a greater level of SCFAs (Liu et al., 2020). These results indicate that, depending on the substrates entering the large intestine, the fermentative profiles of the microbiota are not the same, and this may have an impact on the quantity of SCFAs produced and, therefore, available during exercise. What is more, high levels of starch fermentation can lead to digestive disorders that are detrimental to the health of the host (Garber et al., 2020).

### 4.4 Limitations

This preliminary study provides interesting results but must be interpreted with caution due to several limitations. The number of horses included was limited, as we selected a population from a single training center to minimize environmental variables affecting large intestine microbiota and performance. Additionally, while concentrate feed consumption was assessed individually, hay consumption could only be estimated since the horses had free access to paddocks and stalls. As hay is the main source of dietary fiber, a more controlled assessment of ingested quantities would be necessary to ensure consistent effects on fibrolytic function. Finally, the running test used only running speed as the output measure, as this was an exploratory study. Including additional parameters, such as oxygen consumption, would help better characterize the physiological factors underlying performance and provide further insights into the potential relation between large intestine microbiota and performance.

## 5 CONCLUSION

In conclusion, this pilot study highlights the potential contribution of large intestine microbiota in influencing the exercise capacity of a population of equine athletes subjected to similar management. The fibrolytic efficiency of the large intestine microbiota, rather than its composition alone, appears to be a crucial determinant of exercise capacity by providing different molecules beneficial to host physiology. However, these results provide only a correlation analysis giving no causal link, and further work will be required to elucidate the role of fibroslytic large intestine microbiota on exercise capacity. Furthermore, these findings should be generalized with caution when applied to human athletes, as the contribution of large intestine microbiota to energy requirements in humans is less significant than in horses. Additionally, the physiological determinant of performance may differ between horses and humans. Human athletes often consume large amounts of rapidly digestible carbohydrates while avoiding fibers, which can contribute to an imbalance in the large intestine microbiota (Álvarez‐Herms, 2024). Promoting a healthy digestive environment by providing substrates in line with an individual's digestive capacity could become a future performance‐enhancing strategy for athletes, as this would optimize the microbiota's ability to break down fibers.

## AUTHOR CONTRIBUTIONS

M.V., R.L., S.J., and P.G. conceived and designed research; M.V. and P.G. performed experiments and analyzed data; M.V., R.L., S.J., and P.G. interpreted results of experiments; M.V. prepared figures; M.V. draft manuscript; M.V., R.L., S.J.; N.L., and P.G. edited and revised manuscript; M.V., R.L., S.J., N.L., and P.G. approved final version of the manuscript.

## FUNDING INFORMATION

This research work was supported by the National Association for Research and Technology (ANRT, 2021/1600) and Hunter Valley stable.

## CONFLICT OF INTEREST STATEMENT

No conflicts of interest, financial or otherwise, are declared by the authors.

## ETHICS STATEMENT

This study was conducted under the agreement of the Plateforme NSP Animal Welfare Body (EU0437, #PNSP2201). This protocol was approved by the trainer, and written consent from horse owners was obtained before the sample collection. All procedures were done with respect to animal welfare by an equine veterinary practitioner from the training center.

## Supporting information


Table S1.

Table S2.

## Data Availability

Source data are available upon request to the corresponding author.
