# Supplementary material for: Fibrolytic efficiency of the large intestine microbiota may benefit running speed in French trotters: A pilot study
Source: Physiol Rep. 2024 Nov 12;12(21):e70110. doi: 10.14814/phy2.70110 (PMC11557442; doi:10.14814/phy2.70110)
Supplement: Supplementary file 1 — Table S1. [file PHY2-12-e70110-s002.docx]

Supplementary Table S1 : correlation between relative abundance of Phylum, Family and Genera taxonomic levels and Maximal Running Speed

| **Phylum** | **Family** | **Genera** | **r** | ***p*** | **Mean relative abundance ± SD** |
| --- | --- | --- | --- | --- | --- |
| Actinobacteriota | | | 0.09 | 0.80 | 1.20 ± 0.69 |
|  | Coriobacteriales_Incertae_Sedis | | 0.11 | 0.95 | 0.35 ± 0.31 |
|  |  | Phoenicibacter | 0.30 | 0.68 | 0.18 ± 0.16 |
|  | Eggerthellaceae | | 0.07 | 0.98 | 0.74 ± 0.38 |
| Bacteroidota | | | 0.29 | 0.45 | 33.39 ± 6.2 |
|  | Bacteroidales_RF16_group | | -0.22 | 0.84 | 0.29 ± 0.26 |
|  | Bacteroidales_UCG_001 | | -0.09 | 0.96 | 1.48 ± 0.90 |
|  | F082 | | 0.16 | 0.90 | 7.12 ± 7.96 |
|  | Muribaculaceae | | -0.29 | 0.73 | 0.82 ± 0.73 |
|  | p251o5 | | -0.21 | 0.85 | 6.07 ± 3.02 |
|  | Prevotellaceae | | -0.18 | 0.88 | 8.85 ± 3.39 |
|  |  | Alloprevotella | -0.35 | 0.57 | 0.37 ± 0.26 |
|  |  | Prevotella | -0.15 | 0.84 | 1.52 ± 1.15 |
|  |  | Prevotellaceae_UCG_001 | -0.12 | 0.88 | 4.69 ± 3.01 |
|  |  | Prevotellaceae_UCG_003 | -0.50 | 0.33 | 0.81 ± 0.29 |
|  |  | Prevotellaceae_UCG_004 | -0.30 | 0.67 | 0.77 ± 0.35 |
|  | Rikenellaceae | | 0.16 | 0.90 | 7.77 ± 2.66 |
|  |  | dgA_11_gut_group | 0.10 | 0.90 | 0.25 ± 0.17 |
|  |  | hoa5_07d05_gut_group | 0.07 | 0.93 | 0.20 ± 0.15 |
|  |  | Rikenellaceae_RC9_gut_group | 0.21 | 0.78 | 7.31 ± 2.53 |
| Fibrobacterota | | | -0.45 | 0.27 | 0.79 ± 0.80 |
|  | Fibrobacteraceae | | -0.36 | 0.60 | 0.79 ± 0.80 |
|  |  | Fibrobacter | -0.45 | 0.41 | 0.79 ± 0.80 |
| Firmicutes | | | -0.18 | 0.59 | 62.03 ± 6.03 |
|  | Anaerovoracaceae | | -0.06 | 0.98 | 2.70 ± 1.53 |
|  |  | Anaerovorax | -0.41 | 0.48 | 1.21 ± 0.56 |
|  |  | Eubacterium_nodatum_group | 0.17 | 0.82 | 0.12 ± 0.09 |
|  |  | Family_XIII_AD3011_group | 0.30 | 0.67 | 0.50 ± 0.40 |
|  |  | Mogibacterium | 0.10 | 0.89 | 0.59 ± 0.84 |
|  | Christensenellaceae | | 0.10 | 0.95 | 7.61 ± 3.97 |
|  |  | Christensenellaceae_R_7_group | 0.17 | 0.82 | 7.54 ± 3.95 |
|  | Clostridiaceae | | 0.50 | 0.26 | 0.13 ± 0.19 |
|  | Defluviitaleaceae | | -0.15 | 0.90 | 0.35 ± 0.23 |
|  |  | Defluviitaleaceae_UCG_011 | -0.21 | 0.79 | 0.35 ± 0.23 |
|  | Erysipelotrichaceae | | 0.18 | 0.88 | 0.25 ± 0.14 |
|  | Eubacteriaceae | | -0.01 | 0.99 | 0.14 ± 0.13 |
|  |  | Eubacterium | 0.11 | 0.89 | 0.14 ± 0.13 |
|  | Hungateiclostridiaceae | | -0.50 | 0.26 | 0.98 ± 0.51 |
|  |  | Ruminiclostridium | -0.29 | 0.70 | 0.20 ± 0.11 |
|  |  | Saccharofermentans | -0.30 | 0.67 | 0.78 ± 0.46 |
|  | Lachnospiraceae | | -0.35 | 0.63 | 20.71 ± 3.33 |
|  |  | Agathobacter | -0.61 | 0.18 | 0.67 ± 0.22 |
|  |  | Blautia | -0.29 | 0.69 | 0.25 ± 0.10 |
|  |  | Coprococcus | -0.28 | 0.71 | 0.13 ± 0.09 |
|  |  | Eubacterium_hallii_group | -0.17 | 0.82 | 0.28 ± 0.10 |
|  |  | Eubacterium_ruminantium_group | -0.32 | 0.62 | 0.31 ± 0.20 |
|  |  | Lachnoclostridium | -0.38 | 0.52 | 0.26 ± 0.11 |
|  |  | Lachnospiraceae_AC2044_group | -0.48 | 0.36 | 1.75 ± 0.58 |
|  |  | Lachnospiraceae_FCS020_group | -0.37 | 0.54 | 0.15 ± 0.25 |
|  |  | Lachnospiraceae_ND3007_group | 0.20 | 0.80 | 0.29 ± 0.28 |
|  |  | Lachnospiraceae_NK4A136_group | 0.17 | 0.82 | 2.85 ± 1.20 |
|  |  | Lachnospiraceae_UCG_004 | 0.44 | 0.41 | 0.36 ± 0.20 |
|  |  | Lachnospiraceae_UCG_006 | 0.04 | 0.97 | 0.11 ± 0.06 |
|  |  | Lachnospiraceae_UCG_008 | -0.06 | 0.95 | 0.33 ± 0.09 |
|  |  | Lachnospiraceae_UCG_009 | -0.50 | 0.33 | 0.78 ± 0.34 |
|  |  | Lachnospiraceae_XPB1014_group | -0.07 | 0.93 | 4.73 ± 1.59 |
|  |  | Marvinbryantia | 0.01 | 0.99 | 0.82 ± 0.29 |
|  |  | Oribacterium | -0.26 | 0.72 | 0.61 ± 0.21 |
|  |  | Pseudobutyrivibrio | -0.58 | 0.22 | 0.57 ± 0.17 |
|  |  | Roseburia | -0.03 | 0.97 | 0.31 ± 0.17 |
|  | Lactobacillaceae | | -0.21 | 0.85 | 0.61 ± 0.53 |
|  |  | Ligilactobacillus | -0.25 | 0.75 | 0.52 ± 0.48 |
|  | Monoglobaceae | | -0.03 | 0.99 | 0.17 ± 0.08 |
|  |  | Monoglobus | 0.07 | 0.93 | 0.17 ± 0.08 |
|  | Oscillospiraceae | | -0.04 | 0.99 | 16.38 ± 2.76 |
|  |  | NK4A214_group | -0.42 | 0.46 | 7.34 ± 2.33 |
|  |  | UCG_002 | -0.11 | 0.89 | 4.71 ± 2.51 |
|  |  | UCG_005 | 0.19 | 0.81 | 3.69 ± 1.67 |
|  | Ruminococcaceae | | -0.50 | 0.26 | 3.01 ± 1.20 |
|  |  | Candidatus_Soleaferrea | 0.15 | 0.84 | 0.24 ± 0.13 |
|  |  | Ruminococcus | -0.57 | 0.23 | 1.91 ± 1.05 |
|  | Streptococcaceae | | -0.06 | 0.98 | 0.11 ± 0.18 |
|  | UCG_010 | | 0.20 | 0.85 | 2.52 ± 0.66 |
|  | UCG_011 | | 0.02 | 0.99 | 0.18 ± 0.12 |
| Proteobacteria | | | 0.37 | 0.28 | 0.15 ± 0.10 |
| Spirochaetota | | | -0.23 | 0.53 | 1.73 ± 1.00 |
|  | Spirochaetaceae | | -0.10 | 0.95 | 1.73 ± 1.00 |
|  |  | Treponema | -0.23 | 0.76 | 1.73 ± 1.00 |
| Verrucomicrobiota | | | 0.27 | 0.46 | 0.22 ± 0.11 |
