# Supplementary material for: Fibrolytic efficiency of the large intestine microbiota may benefit running speed in French trotters: A pilot study
Source: Physiol Rep. 2024 Nov 12;12(21):e70110. doi: 10.14814/phy2.70110 (PMC11557442; doi:10.14814/phy2.70110)
Supplement: Supplementary file 2 — Table S2. [file PHY2-12-e70110-s001.docx]

Supplementary Table S2 : correlation between metabolic pathways and Maximal Running Speed

| **Metabolic pathways** | | | **r** | ***p*** | **Mean relative abundance ± SD** |
| --- | --- | --- | --- | --- | --- |
| Genetic Information Processing | | |  |  |  |
|  | Folding, sorting and degradation | |  |  |  |
|  |  | Protein export | 0.04 | 0.93 | 1.55 ± 0.03 |
|  |  | RNA degradation | 0.15 | 0.71 | 0.60 ± 0.02 |
|  |  | Sulfur relay system | 0.31 | 0.35 | 1.24 ± 0.05 |
|  | Replication and repair | |  |  |  |
|  |  | Base excision repair | 0.41 | 0.20 | 0.96 ± 0.03 |
|  |  | DNA replication | 0.20 | 0.59 | 1.29 ± 0.02 |
|  |  | Homologous recombination | 0.32 | 0.34 | 1.63 ± 0.03 |
|  |  | Mismatch repair | 0.30 | 0.37 | 1.85 ± 0.03 |
|  |  | Nucleotide excision repair | 0.35 | 0.29 | 0.86 ± 0.02 |
|  | Transcription | |  |  |  |
|  |  | RNA polymerase | 0.18 | 0.64 | 1.48 ± 0.04 |
|  | Translation | |  |  |  |
|  |  | Aminoacyl-tRNA biosynthesis | 0.34 | 0.31 | 1.89 ± 0.04 |
|  |  | Ribosome | 0.46 | 0.13 | 1.77 ± 0.03 |
| Metabolism | | |  |  |  |
|  | Amino acid metabolism | |  |  |  |
|  |  | Alanine, aspartate and glutamate metabolism | -0.07 | 0.88 | 1.84 ± 0.02 |
|  |  | Arginine and proline metabolism | 0.40 | 0.21 | 0.80 ± 0.01 |
|  |  | Cysteine and methionine metabolism | 0.31 | 0.37 | 1.5 ± 0.02 |
|  |  | Glycine, serine and threonine metabolism | 0.29 | 0.40 | 1.21 ± 0.02 |
|  |  | Histidine metabolism | 0.12 | 0.78 | 1.37 ± 0.02 |
|  |  | Lysine biosynthesis | -0.30 | 0.38 | 1.87 ± 0.02 |
|  |  | Lysine degradation | 0.46 | 0.13 | 0.18 ± 0.01 |
|  |  | Phenylalanine metabolism | 0.12 | 0.78 | 0.24 ± 0.01 |
|  |  | Phenylalanine, tyrosine and tryptophan biosynthesis | 0.14 | 0.73 | 1.43 ± 0.02 |
|  |  | Tyrosine metabolism | 0.17 | 0.66 | 0.22 ± 0.01 |
|  |  | **Valine, leucine and isoleucine biosynthesis** | -0.71 | 0.01 | 2.56 ± 0.04 |
|  |  | Valine, leucine and isoleucine degradation | 0.46 | 0.13 | 0.33 ± 0.02 |
|  | Biosynthesis of other secondary metabolites | |  |  |  |
|  |  | Streptomycin biosynthesis | -0.30 | 0.38 | 1.54 ± 0.07 |
|  |  | Tropane, piperidine and pyridine alkaloid biosynthesis | 0.45 | 0.15 | 0.42 ± 0.02 |
|  | Lipid metabolism | |  |  |  |
|  |  | Biosynthesis of unsaturated fatty acids | -0.33 | 0.32 | 0.33 ± 0.19 |
|  |  | Fatty acid biosynthesis | 0.41 | 0.19 | 2.00 ± 0.03 |
|  |  | Fatty acid degradation | 0.20 | 0.60 | 0.32 ± 0.01 |
|  |  | Glycerolipid metabolism | 0.05 | 0.91 | 0.54 ± 0.02 |
|  |  | Glycerophospholipid metabolism | 0.02 | 0.96 | 0.59 ± 0.02 |
|  |  | Secondary bile acid biosynthesis | -0.25 | 0.49 | 0.57 ± 0.09 |
|  | Carbohydrate metabolism | |  |  |  |
|  |  | Amino sugar and nucleotide sugar metabolism | -0.39 | 0.23 | 1.06 ± 0.03 |
|  |  | Ascorbate and aldarate metabolism | 0.02 | 0.97 | 0.18 ± 0.02 |
|  |  | **Butanoate metabolism** | 0.54 | 0.07 | 0.78 ± 0.02 |
|  |  | **C5-Branched dibasic acid metabolism** | -0.55 | 0.06 | 1.79 ± 0.42 |
|  |  | **Citrate cycle (TCA cycle)** | 0.58 | 0.04 | 0.83 ± 0.03 |
|  |  | Fructose and mannose metabolism | -0.41 | 0.19 | 1.01 ± 0.04 |
|  |  | Galactose metabolism | -0.30 | 0.39 | 0.99 ± 0.05 |
|  |  | Glycolysis / Gluconeogenesis | -0.03 | 0.94 | 1.15 ± 0.02 |
|  |  | Glyoxylate and dicarboxylate metabolism | 0.16 | 0.70 | 0.69 ± 0.01 |
|  |  | Inositol phosphate metabolism | -0.25 | 0.49 | 0.13 ± 0.00 |
|  |  | Pentose and glucuronate interconversions | -0.05 | 0.91 | 0.80 ± 0.04 |
|  |  | Pentose phosphate pathway | 0.17 | 0.67 | 2.03 ± 0.05 |
|  |  | **Propanoate metabolism** | 0.56 | 0.05 | 0.74 ± 0.03 |
|  |  | Pyruvate metabolism | 0.43 | 0.17 | 1.33 ± 0.03 |
|  |  | **Starch and sucrose metabolism** | -0.55 | 0.06 | 1.13 ± 0.04 |
|  | Energy metabolism | |  |  |  |
|  |  | **Carbon fixation pathways in prokaryotes** | 0.63 | 0.02 | 1.07 ± 0.03 |
|  |  | Methane metabolism | -0.07 | 0.88 | 0.58 ± 0.01 |
|  |  | Nitrogen metabolism | 0.07 | 0.88 | 0.60 ± 0.01 |
|  |  | **Oxidative phosphorylation** | -0.51 | 0.09 | 0.44 ± 0.01 |
|  |  | Sulfur metabolism | -0.13 | 0.75 | 0.76 ± 0.01 |
|  | Glycan biosynthesis and metabolism | |  |  |  |
|  |  | Lipopolysaccharide biosynthesis | -0.22 | 0.56 | 0.15 ± 0.05 |
|  |  | Other glycan degradation | -0.23 | 0.54 | 0.60 ± 0.07 |
|  |  | Peptidoglycan biosynthesis | 0.35 | 0.29 | 2.11 ± 0.03 |
|  | Metabolism of cofactors and vitamins | |  |  |  |
|  |  | Biotin metabolism | -0.24 | 0.52 | 0.93 ± 0.04 |
|  |  | Folate biosynthesis | -0.05 | 0.91 | 0.73 ± 0.04 |
|  |  | Lipoic acid metabolism | 0.27 | 0.45 | 0.70 ± 0.12 |
|  |  | Nicotinate and nicotinamide metabolism | 0.28 | 0.42 | 1.14 ± 0.02 |
|  |  | One carbon pool by folate | 0.33 | 0.32 | 1.81 ± 0.04 |
|  |  | Pantothenate and CoA biosynthesis | -0.02 | 0.97 | 1.93 ± 0.05 |
|  |  | Riboflavin metabolism | 0.13 | 0.75 | 0.87 ± 0.04 |
|  |  | **Thiamine metabolism** | 0.56 | 0.05 | 1.85 ± 0.07 |
|  |  | Vitamin B6 metabolism | 0.28 | 0.42 | 1.04 ± 0.03 |
|  | Metabolism of other amino acids | |  |  |  |
|  |  | Glutathione metabolism | 0.11 | 0.79 | 0.34 ± 0.01 |
|  |  | Phosphonate and phosphinate metabolism | -0.22 | 0.56 | 0.15 ± 0.01 |
|  |  | Selenocompound metabolism | 0.06 | 0.90 | 1.08 ± 0.02 |
|  |  | Taurine and hypotaurine metabolism | -0.16 | 0.69 | 0.51 ± 0.21 |
| Organismal Systems | | |  |  |  |
|  | Endocrine system | |  |  |  |
|  |  | Insulin signaling pathway | -0.47 | 0.13 | 0.14 ± 0.01 |
| Metabolism of terpenoids and polyketides | | |  |  |  |
|  |  | Biosynthesis of ansamycins | 0.47 | 0.12 | 6.52 ± 0.23 |
|  |  | Biosynthesis of vancomycin group antibiotics | -0.12 | 0.78 | 2.01 ± 0.14 |
|  |  | **Terpenoid backbone biosynthesis** | 0.54 | 0.07 | 1.52 ± 0.02 |
|  |  | Zeatin biosynthesis | 0.03 | 0.94 | 0.64 ± 0.02 |
|  | Nucleotide metabolism | |  |  |  |
|  |  | Purine metabolism | 0.34 | 0.31 | 0.93 ± 0.01 |
|  |  | Pyrimidine metabolism | 0.46 | 0.13 | 1.25 ± 0.02 |
|  | Xenobiotics biodegradation and metabolism | |  |  |  |
|  |  | Aminobenzoate degradation | 0.41 | 0.20 | 0.12 ± 0.01 |
|  |  | Benzoate degradation | 0.27 | 0.43 | 0.21 ± 0.01 |
|  |  | Nitrotoluene degradation | 0.29 | 0.40 | 0.29 ± 0.03 |

Significant correlations are presented in bold.
